# Supplementary material for: Integrating brainstem and cortical functional architectures
Source: Res Sq. 2023 Nov 28:rs.3.rs-3569352. Preprint. [Version 1] doi: 10.21203/rs.3.rs-3569352/v1 (PMC10705693; doi:10.21203/rs.3.rs-3569352/v1)
Supplement: Supplement 1 [file NIHPPrs3569352v1-supplement-1.pdf]

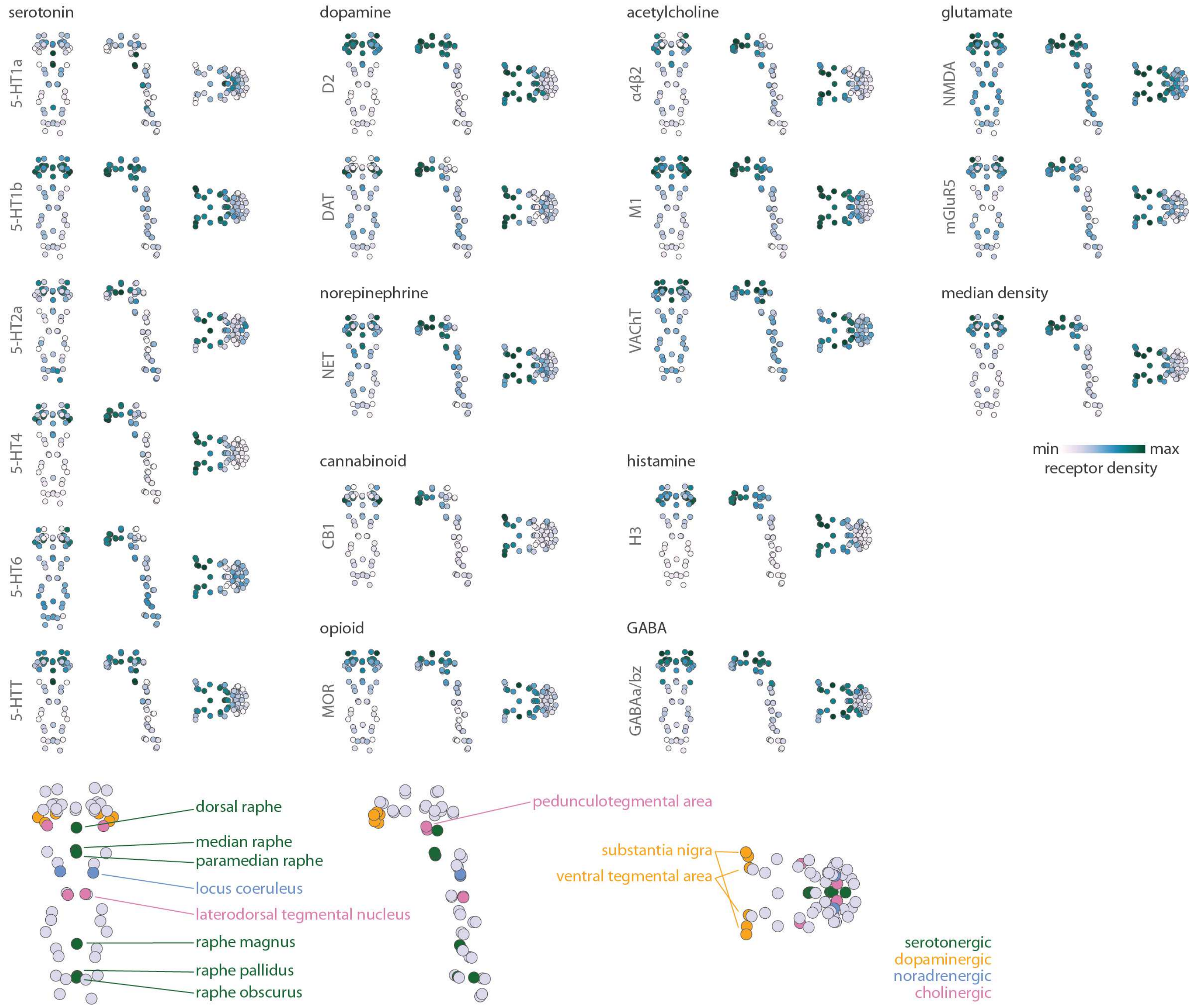

Figure S1. **Neurotransmitter receptor and transporter densities in the brainstem |** 18 PET-derived neurotransmitter receptor and transporter density profiles are shown in the brainstem, as well as the median density across all 18 maps. Coronal (posterior view), sagittal, and axial perspectives of brainstem nuclei are shown. A legend of neuromodulatory nuclei in the brainstem is shown in the bottom row.

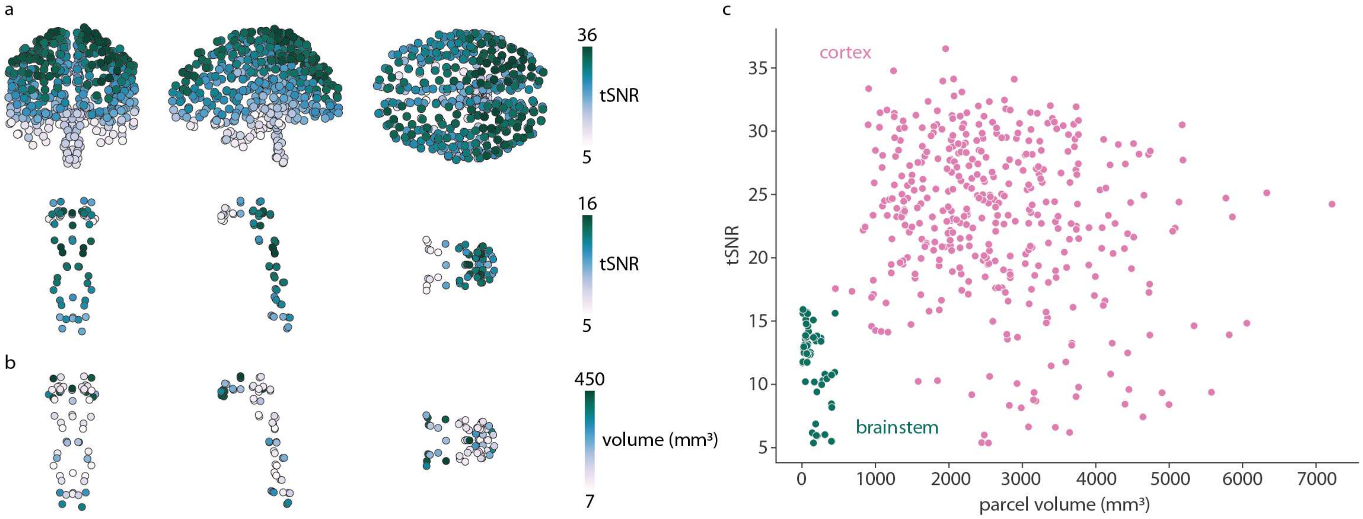

Figure S2. **Temporal signal-to-noise ratio and parcel size.** (a) Temporal signal-to-noise ratio (tSNR) is calculated as the ratio of the mean of a region's time-series to its standard deviation (prior to demeaning the time-series in the preprocessing pipeline). tSNR is shown for the cortex and brainstem together (top) and the brainstem only (bottom). Cortical tSNR $\in [5.37, 36.51]$, brainstem tSNR $\in [5.38, 15.92]$. (b) Parcel volume in mm^3 is shown for each brainstem nucleus. (c) Scatter plot showing the relationship between parcel volume and tSNR of brainstem (green; $r = -0.45$, $p = 0.0004$) and cortical (pink; $r = -0.15$, $p = 0.003$) regions.

**Receptor/ transporter**	**Neurotransmitter**	**Tracer**	**Measure**	N	**References**
D_2	dopamine	[^{11}C]FLB-457	BP_{ND}	55 (26)	[93, 94, 108, 109, 129]
DAT	dopamine	[^{123}I]-FP-CIT	SUVR	174 (109)	[35]
NET	norepinephrine	[^{11}C]MRB	BP_{ND}	77 (50)	[12, 29, 32, 92]
$5\text{-}HT_{1A}$	serotonin	[^{11}C]CUMI-101	BP_{ND}	8 (3)	[13]
$5\text{-}HT_{1B}$	serotonin	[^{11}C]P943	BP_{ND}	23 (15)	[7, 41, 68, 72, 73, 85, 95]
$5\text{-}HT_{2A}$	serotonin	[^{11}C]Cimbi-36	B_{max}	29 (15)	[13]
$5\text{-}HT_4$	serotonin	[^{11}C]SB207145	B_{max}	59 (41)	[13]
$5\text{-}HT_6$	serotonin	[^{11}C]GSK215083	BP_{ND}	30 (30)	[88, 89]
5-HTT	serotonin	[^{11}C]DASB	B_{max}	100 (29)	[13]
$\alpha_4\beta_2$	acetylcholine	[^{18}F]flubatine	V_T	30 (20)	[6, 57]
M_1	acetylcholine	[^{11}C]LSN3172176	BP_{ND}	24 (13)	[74]
VAChT	acetylcholine	[^{18}F]FEOBV	SUVR	18 (5)	[1]
$mGluR_5$	glutamate	[^{11}C]ABP688	BP_{ND}	28 (15)	[34]
$GABA_{A/BZ}$	GABA	[^{11}C]flumazenil	B_{max}	16 (7)	[78]
H_3	histamine	[^{11}C]GSK189254	V_T	8 (7)	[42]
CB_1	cannabinoid	[^{11}C]OMAR	V_T	77 (49)	[36, 76, 79, 90]
MOR	opioid	[^{11}C]carfentanil	BP_{ND}	39 (19)	[117]

TABLE S1. **Neurotransmitter receptors and transporters** | BP_{ND} = non-displaceable binding potential; V_T = tracer distribution volume; B_{max} = density (pmol/ml) converted from binding potential (5-HT) or distributional volume (GABA) using autoradiography-derived densities; SUVR = standard uptake value ratio. Values in parentheses (under N) indicate number of males.

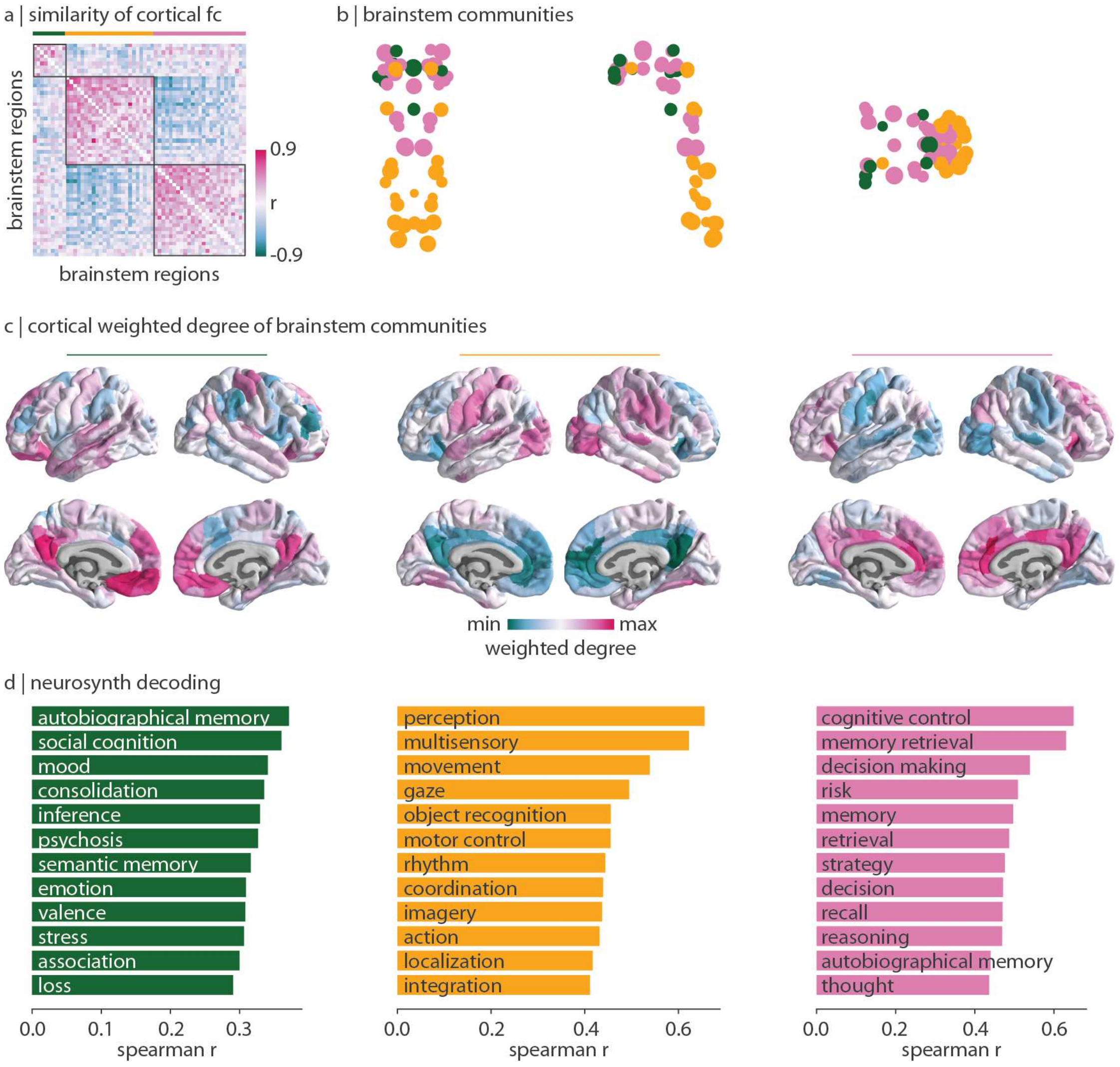

Figure S3. **Brainstem communities when** $\gamma = 1.9$ | The Louvain community detection algorithm was repeated for $\gamma = 1.9$ which identified three stable communities.

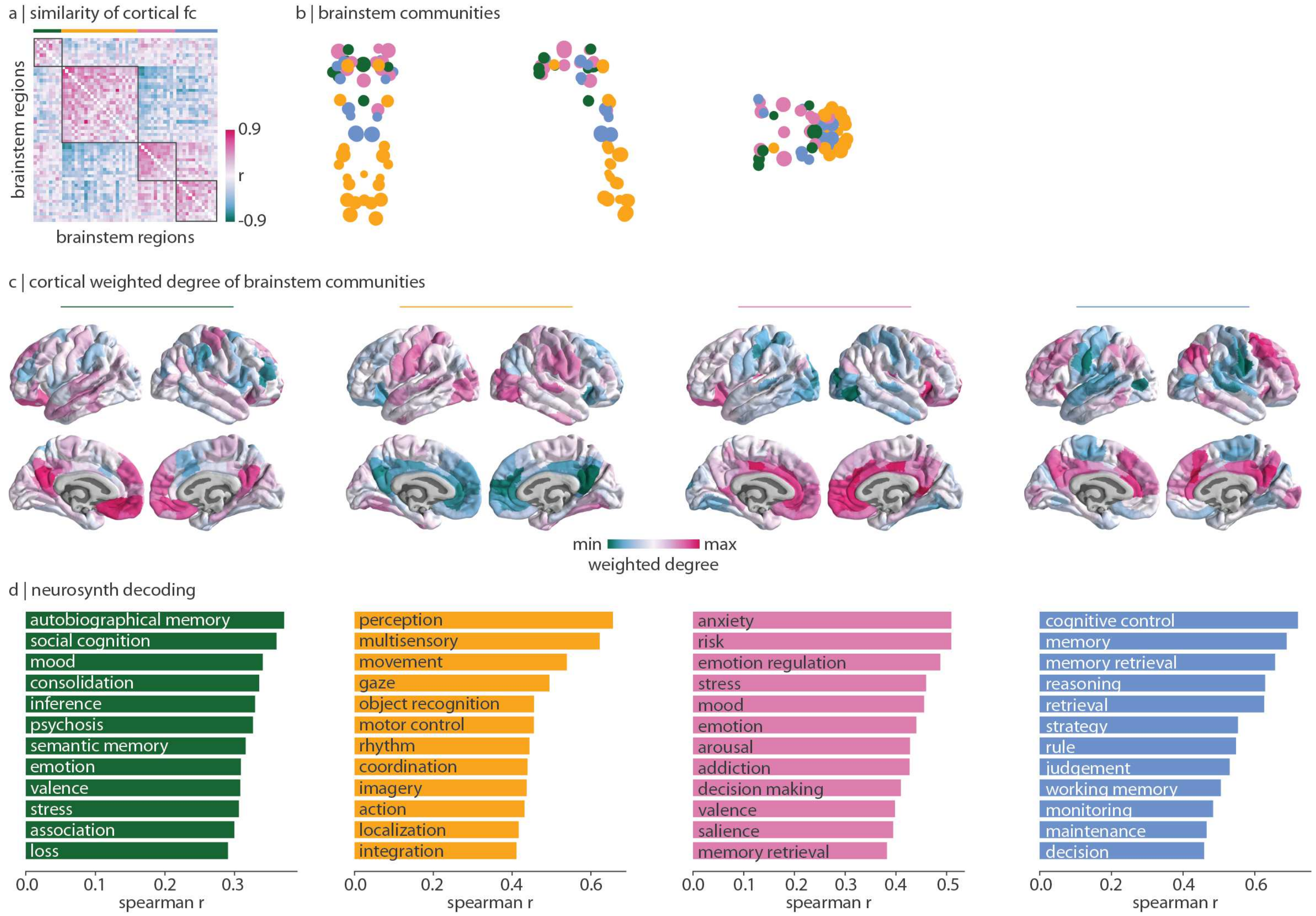

Figure S4. **Brainstem communities when $\gamma = 2.2$** | The Louvain community detection algorithm was repeated for $\gamma = 2.2$ which identified four stable communities.

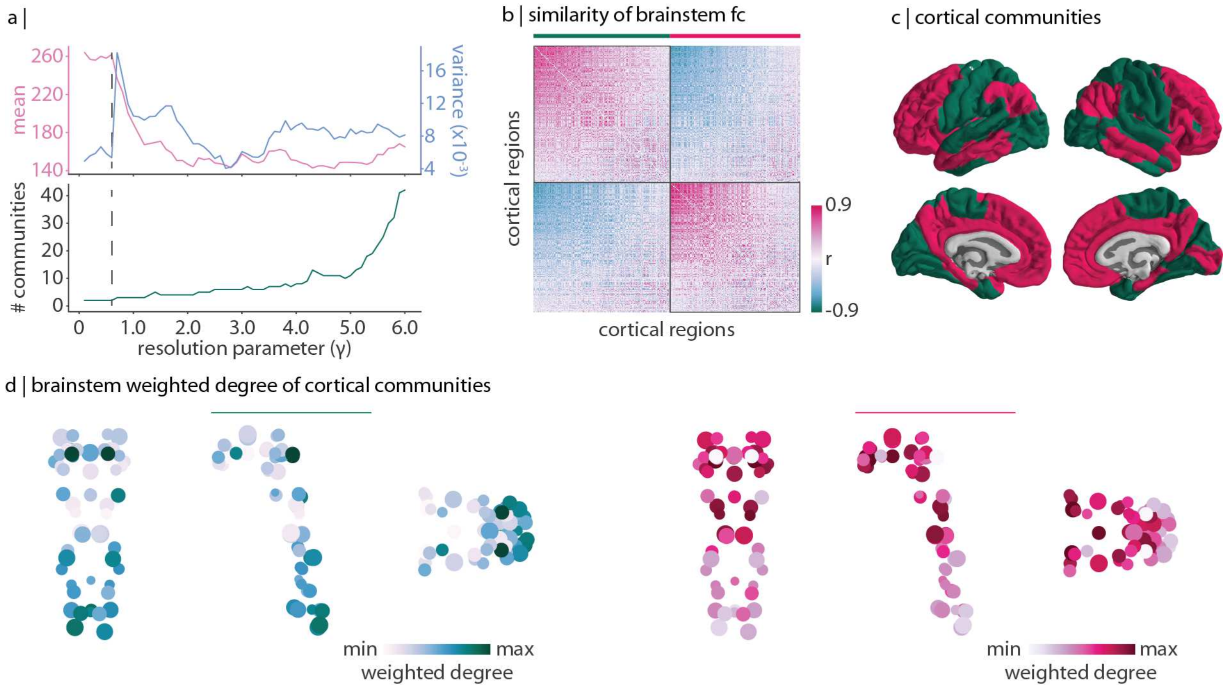

Figure S5. **Cortical communities of brainstem functional connectivity |** The Louvain community detection algorithm was applied to a correlation matrix representing how similarly (Spearman's r) two cortical regions are functionally connected with the brainstem above and beyond the dominant pattern of brainstem connectivity. (a) Top: mean and variance of the z-scored rand index across 250 repetitions of the Louvain algorithm at each resolution parameter $\gamma \in [0.1, 6.0]$. Bottom: number of communities identified for each γ. The dashed vertical line exists at $\gamma = 0.6$. (b) Cortical region $\times$ region correlation matrix representing how similarly cortical regions are functionally connected with the brainstem. Regions are ordered according to the two communities identified at $\gamma = 0.6$. (c) Community affiliations for each cortical region. (d) Brainstem weighted degree of the green (left, unimodal) community and the red (right, transmodal) community. Specifically, for each brainstem nucleus, we sum its regressed functional connectivity with all cortical regions in the green/red community.

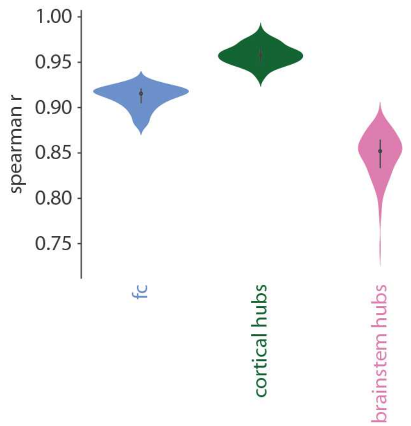

Figure S6. **Split-half analysis |** The 20 participants included in the present study were randomly divided into two groups of 10 (100 repetitions). Group-average functional connectivity, cortex-to-brainstem weighted degree patterns, and brainstem-to-cortex weighted degree patterns were recalculated within these groups are correlated.

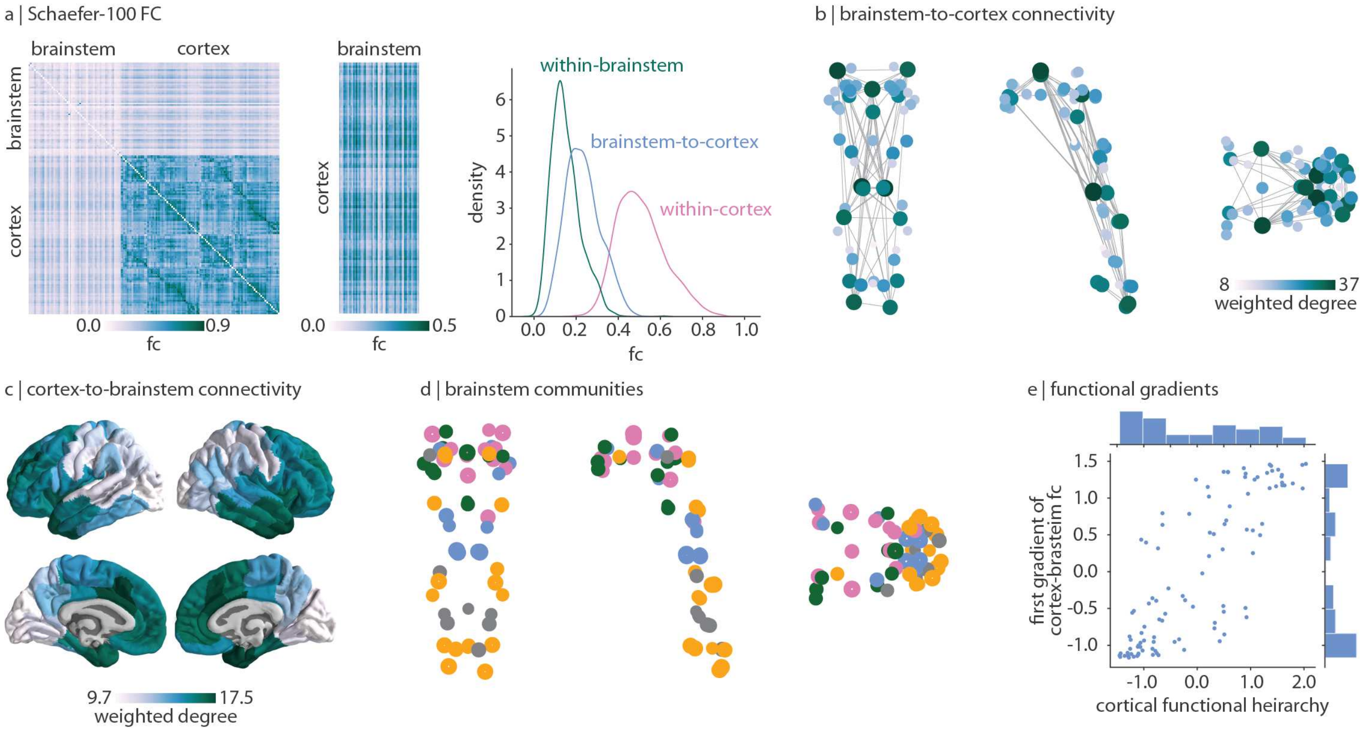

Figure S7. **Replication using 100 cortical regions |** Analyses were repeated using the 100-region Schaefer parcellation [97]. (a) Functional connectivity and functional connectivity density distributions. (b) Brainstem-to-cortex weighted degree. (c) Cortex-to-brainstem weighted degree. (d) Community affiliations of brainstem nodes under identical parameters as shown in Fig. 3. (e) Correlation between the first gradient of cortex-to-brainstem functional connectivity and the cortical functional hierarchy.

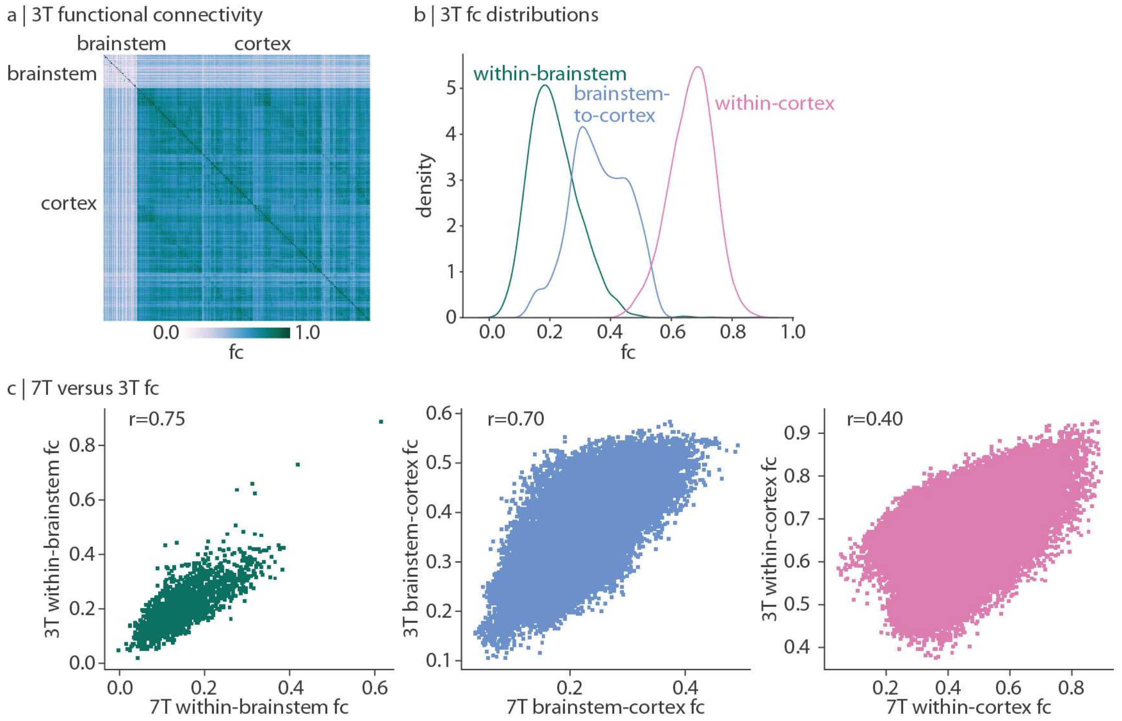

Figure S8. **Replication using 3 Tesla fMRI data |** Analyses were repeated using 3 Tesla fMRI data acquired in the same 20 subjects, under the Schaefer-400 parcellation [97]. (a) Functional connectivity. (b) Functional connectivity density distributions. (c) Spearman correlations between 7 Tesla functional connectivity data used in the main analyses and 3 Tesla functional connectivity replication data.

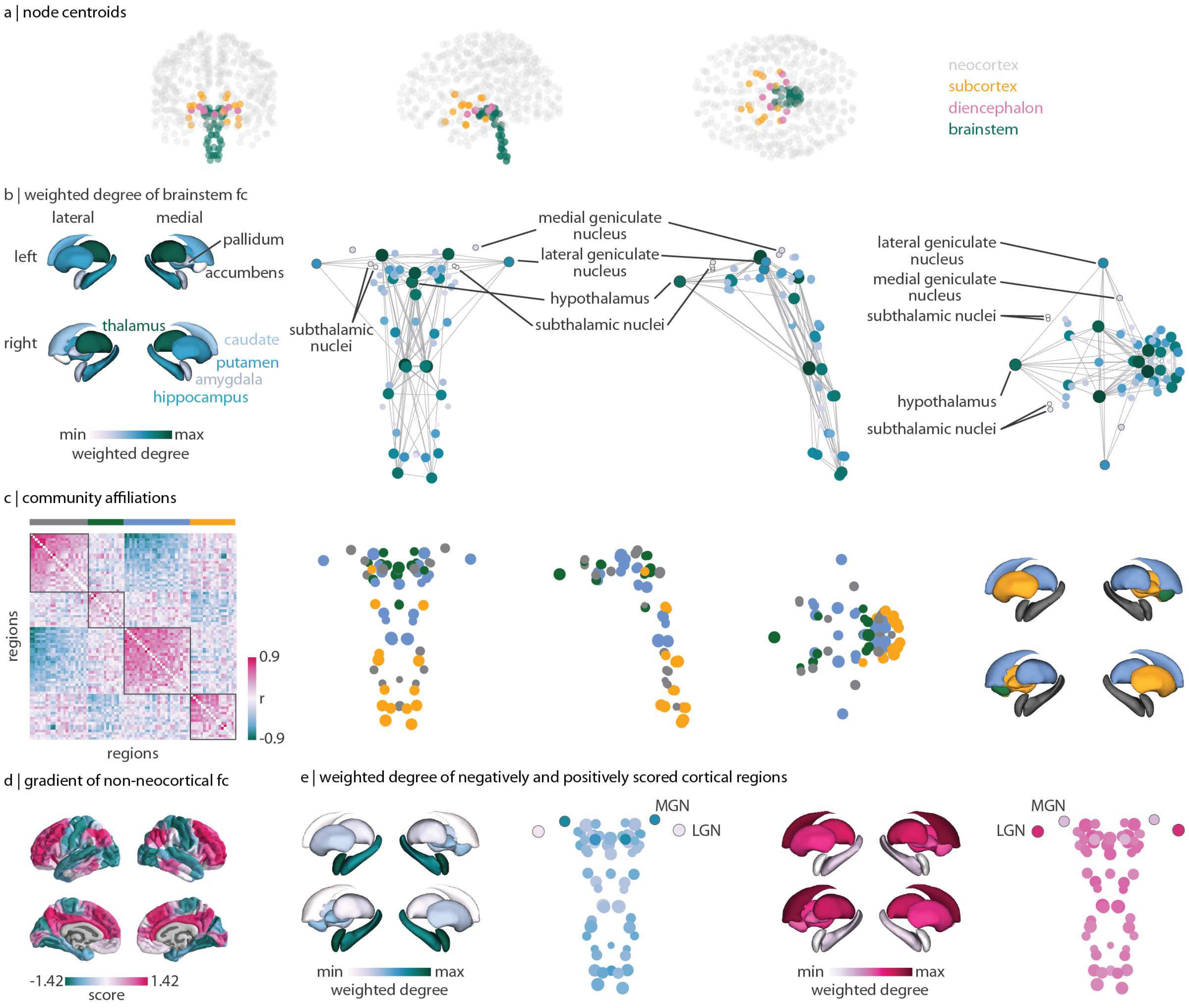

Figure S9. **Extending analyses to subcortical and diencephalic structures** | Functional images were also acquired for the 14 bilateral FreeSurfer subcortical structures (caudate, putamen, pallidum, nucleus accumbens, thalamus, amygdala, hippocampus (not technically subcortex but allocortex)) as well as 8 bilateral diencephalic structures from the Brainstem Navigator (lateral geniculate nucleus, medial geniculate nucleus, subthalamic nuclei subregions 1 & 2). (a) For each brain regions, the centroid coordinate is plotted with colours indicating structure. Grey: 400 neocortical structures; yellow: 14 FreeSurfer subcortical structures; pink: 8 Brainstem Navigator diencephalic regions; green: 58 Brainstem Navigator brainstem nuclei. (b) Left: FreeSurfer subcortical plot of weighted degree of brainstem functional connectivity, representing how much each FreeSurfer subcortical parcel is connected with the brainstem. Each structure is labeled. Right: Brainstem Navigator brainstem and diencephalic centroid coordinates coloured according to their weighted degree of brainstem functional connectivity, representing how much each nucleus is connected with the 58 brainstem structures. The 8 diencephalic nuclei are labeled. (c) Left: region $\times$ region similarity matrix representing how similarly two non-neocortical (i.e. brainstem, subcortical, or diencephalic) regions are functionally connected with the cortex. Outlines are placed around the identified communities. Middle: point brain plot of community assignments for brainstem and diencephalic nuclei. Right: FreeSurfer surface plot of community assignments for FreeSurfer subcortical regions. (d) Left: region $\times$ region similarity matrix representing how similarly two cortical regions are functionally connected with non-neocortical regions (i.e. brainstem, subcortical, diencephalic). Outlines are shown around the seven Yeo-Krienen resting-state networks (order: control, default mode, dorsal attention, limbic, ventral attention, somato-motor, visual). Middle: coritcal surface plot of the first gradient from diffusion map embedding of how similarly cortical regions are functionally connected with non-neocortical regions. Right: FreeSurfer subcortical weighted degree patterns, calculated as the sum of a FreeSurfer subcortical region's functional connectivity with all negatively- (left) or positively- (right) scored regions of the cortical gradient shown on the left. FreeSurfer subcortical structures were plotted using the `enigmatoolbox` [63].

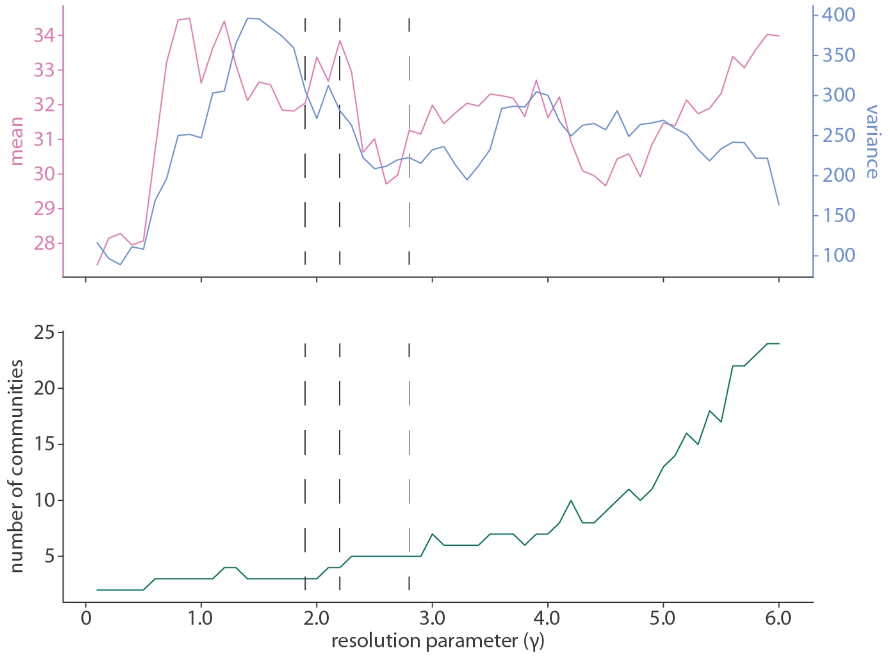

Figure S10. **Community detection performance across resolution parameter γ |** Top: mean and variance of the z-scored rand index across 250 repetitions of the Louvain community detection algorithm at each γ (for $\gamma \in [0.1, 6.0]$). Community detection solutions are considered better quality (i.e. more stable) when the mean of the z-scored rand index is high and the variance is low. Dashed vertical lines are placed at values of γ where the community detection solution is shown in the text ($\gamma = 1.9$ shown in Fig. S3, $\gamma = 2.2$ shown in Fig. S4, and $\gamma = 2.8$ shown in Fig. 3). Bottom: the number of communities identified by the algorithm across values of γ.

action	eating	insight	naming	semantic memory
adaptation	efficiency	integration	navigation	sentence comprehension
addiction	effort	intelligence	object recognition	skill
anticipation	emotion	intention	pain	sleep
anxiety	emotion regulation	interference	perception	social cognition
arousal	empathy	judgment	planning	spatial attention
association	encoding	knowledge	priming	speech perception
attention	episodic memory	language	psychosis	speech production
autobiographical memory	expectancy	language comprehension	reading	strategy
balance	expertise	learning	reasoning	strength
belief	extinction	listening	recall	stress
categorization	face recognition	localization	recognition	sustained attention
cognitive control	facial expression	loss	rehearsal	task difficulty
communication	familiarity	maintenance	reinforcement learning	thought
competition	fear	manipulation	response inhibition	uncertainty
concept	fixation	meaning	response selection	updating
consciousness	focus	memory	retention	utility
consolidation	gaze	memory retrieval	retrieval	valence
context	goal	mental imagery	reward anticipation	verbal fluency
coordination	hyperactivity	monitoring	rhythm	visual attention
decision	imagery	mood	risk	visual perception
decision making	impulsivity	morphology	rule	word recognition
detection	induction	motor control	salience	working memory
discrimination	inference	movement	search	
distraction	inhibition	multisensory	selective attention	

TABLE S2. **Neurosynth terms |** Terms that overlapped between the Neurosynth database [127] and the Cognitive Atlas [87] were used in the cognitive decoding analysis.
